# Supplementary material for: A mosaic monoploid reference sequence for the highly complex genome of sugarcane
Source: Nat Commun. 2018 Jul 6;9:2638. doi: 10.1038/s41467-018-05051-5 (PMC6035169; doi:10.1038/s41467-018-05051-5)

# Supplementary Information

## A mosaic monoploid reference sequence for the highly complex genome of sugarcane

**Garsmeur *et al*.**

**Supplementary Table 1.**
Selection, sequencing and contig assembly of sugarcane BACs

| **Sugarcane BAC selection based on sorghum through WGP** | | | | **577 available BAC sequences** | | | | | **PACBio Sequencing 4,083 BACs** | | | | | **Total BACs sequenced** |
|---|---|---|---|---|---|---|---|---|---|---|---|---|---|---|
| Sorghum chromosome | Nb of WGP-tags mapped | Nb of BACs anchored | MTP of BACs | Nb of BACs | Assembly (contig number) | | | | Nb of BAC | Assembly (contig number) | | | | |
| | | | | | 1 | 2 | 3 | ≥4 | | 1 | 2 | 3 | ≥4 | |
| Sb01 | 20802 | 1924 | 811 | 104 | 85 | 4 | 9 | 6 | 674 | 585 | 59 | 20 | 10 | 778 |
| Sb02 | 15388 | 1598 | 602 | 79 | 65 | 3 | 5 | 6 | 515 | 429 | 62 | 9 | 15 | 594 |
| Sb03 | 16144 | 1624 | 654 | 97 | 78 | 6 | 5 | 8 | 537 | 443 | 66 | 18 | 10 | 634 |
| Sb04 | 12871 | 1261 | 505 | 58 | 49 | 1 | 0 | 8 | 438 | 368 | 47 | 13 | 10 | 496 |
| Sb05 | 6167 | 827 | 298 | 28 | 20 | 5 | 0 | 3 | 261 | 222 | 26 | 8 | 5 | 289 |
| Sb06 | 9894 | 1060 | 414 | 57 | 44 | 3 | 7 | 3 | 347 | 317 | 25 | 3 | 2 | 404 |
| Sb07 | 7448 | 871 | 313 | 32 | 27 | 1 | 0 | 4 | 273 | 233 | 29 | 7 | 4 | 305 |
| Sb08 | 5691 | 623 | 274 | 24 | 18 | 3 | 2 | 1 | 241 | 212 | 19 | 5 | 5 | 265 |
| Sb09 | 8732 | 891 | 399 | 51 | 45 | 1 | 3 | 2 | 340 | 289 | 30 | 13 | 8 | 391 |
| Sb10 | 10115 | 1053 | 390 | 47 | 45 | 0 | 0 | 2 | 332 | 277 | 42 | 9 | 4 | 379 |
| | 113252 | 11732 | **4660** | 577 | 476 (82%) | 27 (5%) | 31 (5%) | 43 (7%) | 3958 | 3375 (85%) | 405 (10%) | 105 (3%) | 73 (2%) | **4535** |

**Supplementary Table 2.**

Metrics of sugarcane and sorghum predicted gene models.

*For sugarcane, mono-exonic genes without IPR domains and no hits on Uniprot/Trembl were not taken into account for the metrics. For sorghum, version V3.1 was used and if several alternative transcripts were predicted, the longuest was kept

| | Sugarcane STP | Sorghum |
|---|---|---|
| **Assembly** | | |
| length (Mb) | 382 | 732 |
| **Gene** | | |
| Total number of genes* | 22,780 (out of 25, 316) | 24,960 (out of 34,129) |
| Mean length of predicted polypeptides (bp) | 2,119 | 2,974 |
| **Exon** | | |
| Mean/median number per gene | 4 / 3 | 4.9 / 3 |
| Mean length (bp) | 281 | 248 |
| Max length (bp) | 6,018 | 7,856 |
| **Introns** | | |
| mean length (bp) | 391 | 433 |
| max length (bp) | 16,658 | 18,858 |

**Supplementary Table 3.**

Interpro domains (IPRs) represented in sugarcane genes that were not found in the sorghum genome. *The same gene can contain several IPRs. The table presents only IPRs found in at least 10 genes

| InterPro domain | Info | Nb of genes* |
|---|---|---|
| IPR032675 | Leucine-rich repeat domain superfamily | 175 |
| IPR001810 | F-box domain | 139 |
| IPR027417 | P-loop containing nucleoside triphosphate hydrolase | 71 |
| IPR001128 | Cytochrome P450 | 61 |
| IPR002182 | NB-ARC | 54 |
| IPR011991 | ArsR-like helix-turn-helix domain | 46 |
| IPR025315 | Domain of unknown function DUF4220 | 38 |
| IPR000719 | Protein kinase domain | 37 |
| IPR001611 | Leucine-rich repeat | 36 |
| IPR011009 | Protein kinase-like domain superfamily | 35 |
| IPR008974 | TRAF-like | 34 |
| IPR007658 | Protein of unknown function DUF594 | 33 |
| IPR011333 | SKP1/BTB/POZ domain superfamily | 32 |
| IPR000210 | BTB/POZ domain | 30 |
| IPR012871 | Protein of unknown function DUF1677, Oryza sativa | 29 |
| IPR013083 | Zinc finger, RING/FYVE/PHD-type | 28 |
| IPR025287 | Wall-associated receptor kinase, galacturonan-binding domain | 25 |
| IPR003591 | Leucine-rich repeat, typical subtype | 25 |
| IPR013210 | Leucine-rich repeat-containing N-terminal, plant-type | 24 |
| IPR034090 | BPM, C-terminal | 21 |
| IPR002213 | UDP-glucuronosyl/UDP-glucosyltransferase | 20 |
| IPR017441 | Protein kinase, ATP binding site | 19 |
| IPR029058 | Alpha/Beta hydrolase fold | 18 |
| IPR017972 | Cytochrome P450, conserved site | 18 |
| IPR008271 | Serine/threonine-protein kinase, active site | 18 |
| IPR017451 | F-box associated interaction domain | 17 |
| IPR004140 | Exocyst complex component Exo70 | 17 |
| IPR001841 | Zinc finger, RING-type | 17 |
| IPR006566 | FBD domain | 16 |
| IPR011676 | Domain of unknown function DUF1618 | 15 |
| IPR016159 | Cullin repeat-like-containing domain superfamily | 14 |
| IPR016024 | Armadillo-type fold | 14 |
| IPR003480 | Transferase | 14 |
| IPR002083 | MATH/TRAF domain | 14 |
| IPR002016 | Haem peroxidase, plant/fungal/bacterial | 14 |
| IPR020683 | Ankyrin repeat-containing domain | 13 |
| IPR016140 | Bifunctional inhibitor/plant lipid transfer protein/seed storage helic | 13 |
| IPR013783 | Immunoglobulin-like fold | 13 |
| IPR005174 | Domain unknown function DUF295 | 13 |
| IPR016040 | NAD(P)-binding domain | 12 |
| IPR011989 | Armadillo-like helical | 12 |
| IPR002110 | Ankyrin repeat | 12 |
| IPR001881 | EGF-like calcium-binding domain | 12 |
| IPR020846 | Major facilitator superfamily domain | 11 |
| IPR018097 | EGF-like calcium-binding, conserved site | 11 |
| IPR015943 | WD40/YVTN repeat-like-containing domain superfamily | 11 |
| IPR013320 | Concanavalin A-like lectin/glucanase domain superfamily | 11 |
| IPR000742 | EGF-like domain | 11 |
| IPR000152 | EGF-type aspartate/asparagine hydroxylation site | 11 |
| IPR013830 | SGNH hydrolase-type esterase domain | 10 |
| IPR010255 | Haem peroxidase | 10 |
| IPR003018 | GAF domain | 10 |
| IPR001220 | Legume lectin domain | 10 |

**Supplementary Figure 1.**

Insertion date of full length LTR retrotransposons. The number of complete TEs for each family is indicated in brackets.

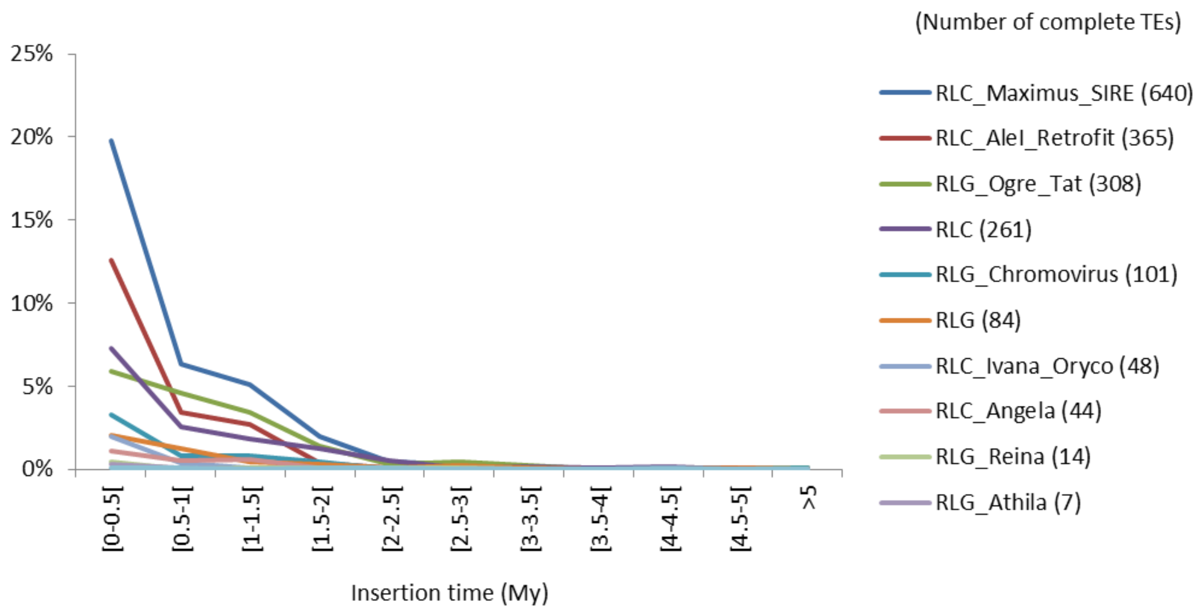

**Supplementary Figure 2**

Circos visualizations represent orthologous relationships between the 132 CGs of the R570 sugarcane cultivar genetic map and the 10 sorghum chromosomes (Sb1 to Sb10) with alignment of 5,406 markers from the R570 genetic map on sorghum (grey and color links). Based on the alignment for each sugarcane CG of a majority of the markers (color links) to one (a) or two (b) sorghum chromosomes, R570 CGs were assembled in hom(oe)ology groups (HG).

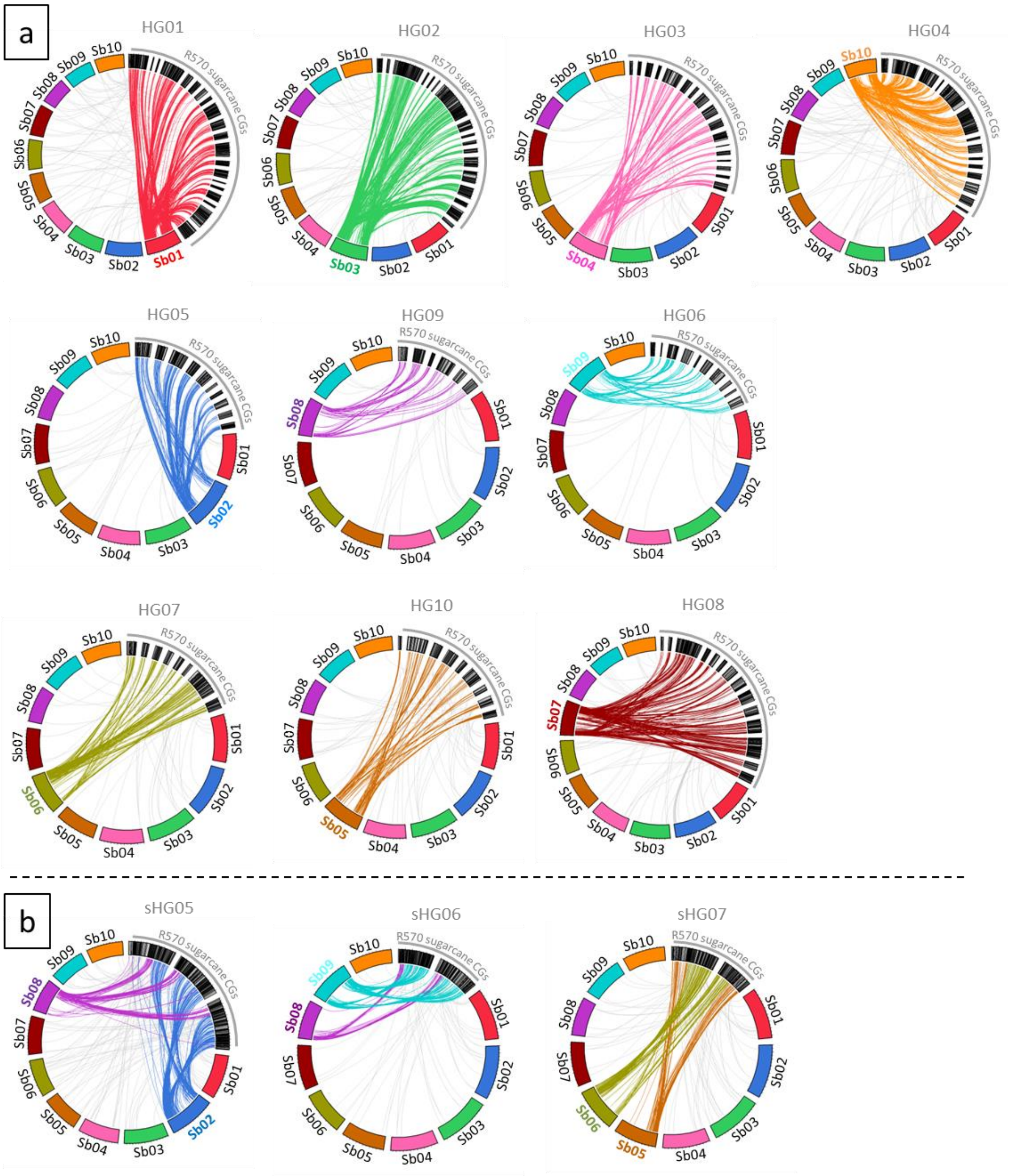

**Supplementary Figure 3**

A total 94 individuals from a mapping population derived from a R570 x MQ76-53 cross described in Raboin et al. (2006) were genotyped with GBS using the Pst1 restriction enzyme. Raw sequencing and map construction were performed as described for the R570 mapping population, except that single dose SNP markers were selected based on an expected segregation ratio of 1:1 (test $\chi 2$, p=0.05). A total of 11,612 single dose SNPs were used to build the MQ76-53 map. Orthologous relationships with sorghum chromosomes were analyzed as described for the R570 map using 7,401 sugarcane GBS markers that could be aligned with the sorghum sequence. 86 CGs had a large majority of markers aligned with one single sorghum chromosome (not shown), while for 18 CGs a large majority of markers aligned with two distinct sorghum chromosomes.

Circos visualizations represent orthologous relationships between these 18 CGs and sorghum chromosomes with marker alignments from the MQ76-53 genetic map on sorghum (grey and color links). Note that MQ76-53 is an old Australian sugarcane clone derived from a cross between the old Trojan cultivar and the S. spontaneum SES528 clone. Its genetic structure should therefore be close to that of an interspecific F1 and thus a higher number of CGs originating from S. spontaneum is expected in this clone as compared to the R570 cultivar.

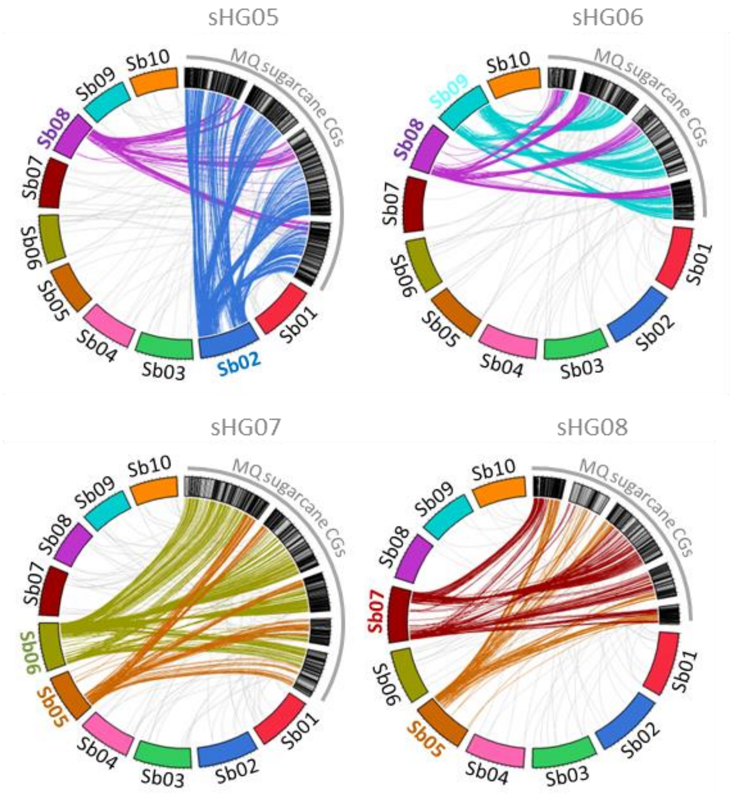

Supplement: Supplementary file 1 — Supplementary Information [file 41467_2018_5051_MOESM1_ESM.pdf]
